# TNFα-senescence initiates a STAT-dependent positive feedback loop, leading to a sustained interferon signature, DNA damage, and cytokine secretion

**DOI:** 10.18632/aging.101328

**Published:** 2017-11-22

**Authors:** Renuka Kandhaya-Pillai, Francesc Miro-Mur, Jaume Alijotas-Reig, Tamara Tchkonia, James L. Kirkland, Simo Schwartz

**Affiliations:** ^1^ Aging Basic Research Group, Molecular Biology and Biochemistry Research Center for Nanomedicine, CIBBIM-Nanomedicine, Vall d'Hebron University Hospital, Research Institute (VHIR), Barcelona, Spain; ^2^ Networking Research Center on Bioengineering, Biomaterials, and Nanomedicine (CIBER-BBN), 08035, Barcelona, Spain; ^3^ Robert and Arlene Kogod Center on Aging, Mayo Clinic, Rochester, MN 55905, USA

**Keywords:** senescence, inflammation, DNA-damage, interferon response genes, JAK/STAT pathway

## Abstract

Cellular senescence is a cell fate program that entails essentially irreversible proliferative arrest in response to damage signals. Tumor necrosis factor-alpha (TNFα), an important pro-inflammatory cytokine secreted by some types of senescent cells, can induce senescence in mouse and human cells. However, downstream signaling pathways linking TNFα-related inflammation to senescence are not fully characterized. Using human umbilical vein endothelial cells (HUVECs) as a model, we show that TNFα induces permanent growth arrest and increases p21^CIP1^, p16^INK4A^, and SA-β-gal, accompanied by persistent DNA damage and ROS production. By gene expression profiling, we identified the crucial involvement of inflammatory and JAK/STAT pathways in TNFα-mediated senescence. We found that TNFα activates a STAT-dependent autocrine loop that sustains cytokine secretion and an interferon signature to lock cells into senescence. Furthermore, we show STAT1/3 activation is necessary for cytokine and ROS production during TNFα-induced senescence. However, inhibition of STAT1/3 did not rescue cells from proliferative arrest, but rather suppressed cell cycle regulatory genes and altered TNFα-induced senescence. Our findings suggest a positive feedback mechanism *via* the STAT pathway that sustains cytokine production and reveal a reciprocal regulatory role of JAK/STAT in TNFα-mediated senescence.

## INTRODUCTION

Cellular senescence is a dynamic tumor suppression mechanism that limits the proliferation of impaired cells by enforcing stable cell cycle arrest [1]. Normal cells eventually enter stable proliferative arrest after repeated cell divisions, termed replicative senescence [2]. Senescence can be triggered by diverse forms of stress stimuli, including telomere dysfunction [3], DNA damage signals [4–6], and chronic oxidative stress, such as that induced by exposure to sub-cytotoxic concen-trations of t-BHP, ethanol, or H_2_O_2_ [7, 8]. Senescent cells remain metabolically active and often display altered morphology, including multi-nucleation [9, 10]. Markers of cellular senescence include functionally activated p53- and p16^INK4A^ (p16)-related tumor suppressor pathways, increased cellular senescence-associated β-galactosidase activity (SA-β-gal), and increased expression of CDK inhibitors, including p15^INK4B^, p16, and p21^CIP1^ (p21) [1, 10–12]. Formation of senescence-associated heterochromatic foci (SAHF) and chromatin remodeling epigenetically regulate senescence by repressing transcription of genes involved in proliferation [13, 14]. Senescent cells can secrete a wide range of growth factors, cytokines, chemokines, extracellular matrix proteases, and degra-dative enzymes known as the senescence-associated secretory phenotype (SASP) or senescence messaging secretome [15–18]. Cytokines, such as interleukin-6 (IL6), IL8, and IL1α, are SASP components that can promote or sustain senescence [17, 19–22]. Consequent-ly, interplay between persistent DNA damage responses and SASP elements is enforced upon establishment of senescence [23–25].

Engagement of senescence appears to require integration of multiple signaling pathways. However, the diverse pathways influencing this complex process seem to converge on two tumor suppressor pathways, which include p53 and p16. Mitogen Activated Protein Kinase (MAPK) pathways (ERK, JNK, and p38) are also known to be implicated in senescence [26]. In particular, activation of p38 MAPK has been reported to play a significant role in stress-induced senescence [27–29]. In addition, cytokine signaling pathways, such as NF-κB, Ras-MEF-ERK, and Janus Kinase (JAK)/ signal transducers and activators of transcription (STAT), are among pathways that contribute to senescence and the SASP [30–34]. Despite recent advances in unraveling molecular mechanisms of senescence, complex changes in gene expression and multiple regulatory events complicate interpretation of this phenomenon.

Persistent presence of low level circulating cytokines eventually leads to chronic activation of the immune system, termed inflamm-aging. Chronic inflammation and immune system remodeling appear to be related to aging and various age-related pathologies, such as arthritis, atherosclerosis, cancer, and diabetes [35]. However, it has become evident that direct links exist among cellular aging, inflammation, and age-related dysfunction [34, 36, 37]. Senescent cells act as a source of chronic inflammation due to SASP factors, such as inflammatory cytokines and chemokines, which are mediators of inflammation and intracellular signaling [1, 15, 26, 38]. However, the mechanisms responsible for initiating and sustaining senescence-associated secretory patterns are not completely understood.

Tumor necrosis factor-alpha (TNFα) is a multi-functional pro-inflammatory cytokine known to mediate a broad range of biological functions, including cell proliferation, differentiation, and inflammation [39]. TNFα acts as a potent inducer of inflammatory processes and plays a crucial role in the pathogenesis of numerous chronic inflammatory diseases [40]. Senescent cells can be a source of chronic inflammation due to secretion of in-flammatory chemokines and cytokines, including TNFα [34]. In the present study, we investigated signal trans-duction events linked to senescence mediated by TNFα.

## RESULTS

### TNFα induces senescence in HUVECs with continually elevated ROS and DNA damage

**Figure 1:**
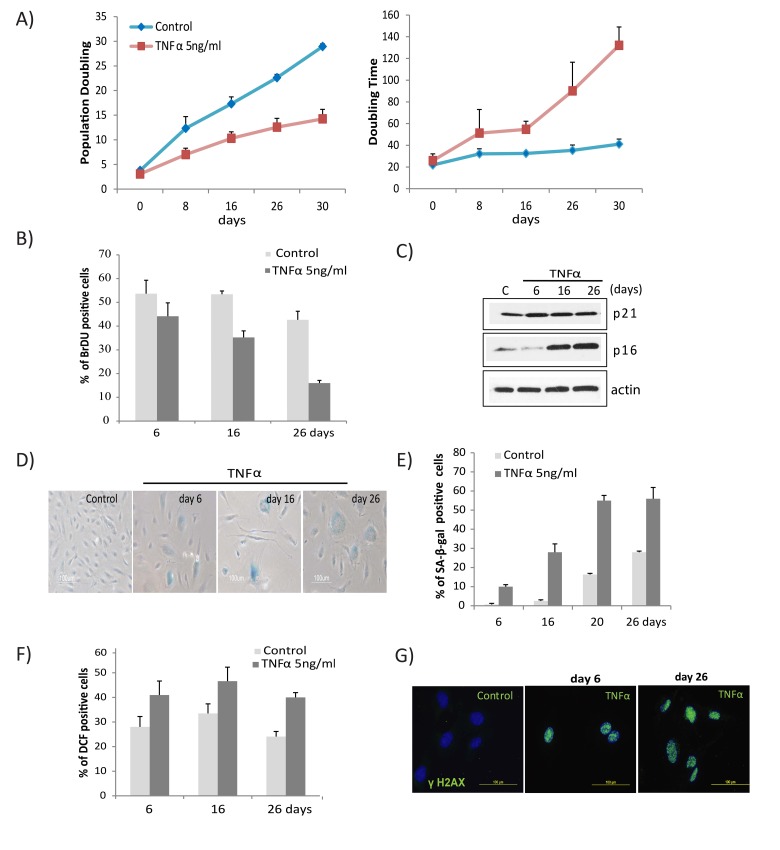
TNFα induces senescence and DNA damage in HUVECs (**A**) Long-term growth curve of cells exposed to recombinant human TNFα (5ng/ml). Untreated cells were used as controls. Population doubling and doubling times were calculated based on cell density at confluence. Data represent mean values from 3 independent experiments. (**B**) The percentage of BrdU-positive cells was determined by FACS analysis in cells untreated or chronically treated with TNFα at the concentration indicated. (**C**) Western blot analysis of p21, p16, and actin in cells treated with TNFα 5ng/ml for the indicated times. (**D**) SA-β-gal activity in TNFα (5ng/ml)-treated or control cells for the indicated number of days. (**E**) Percentages of SA-β-gal-positive cells in control or TNFα-treated cultures. The data represent 2 independent counts of 200 cells from 3 independent experiments. (**F**) Intracellular ROS levels were monitored by 2′,7′-dichlorodihydrofluorescein diacetate staining followed by flow cytometry. Bar graph represents percentage of DCFDA-positive cells treated with TNFα or medium alone. (**G**) Immunofluorescence detection of γH2AX foci in controls or cells treated with TNFα (5ng/ml) for indicated days. Data in **A**, **B**, **E**, and **F** represent mean value ± standard deviation (s.d.) from n=3, 2, 3, and 2 independent experiments, respectively.

To determine the long term effects of the pro-inflammatory cytokine TNFα on endothelial cells, HUVECs were chronically exposed to medium alone or different concentrations of TNFα. Cells exposed to TNFα exhibited a dose- and time-dependent decrease in proliferative rate, as apparent from a linear decrease in population doublings with an increase in doubling time (Fig. 1A, Fig. S1A). Cell cycle analysis revealed prolonged exposure to TNFα significantly impaired proliferation of cells, as evidenced by progressive decline in abundance of BrdU-incorporating cells (Fig.1B) accompanied by a decreased percentage of S-phase and increased G1/G2-phase cells compared to control (Fig. S1B), indicating proliferative arrest. Cells exposed to TNFα also developed classic senescent phenotypes, including a flattened, multi-nucleated, giant cell morphology (Fig. S1C), and concomitantly in-creased expression of senescence markers, including p16, p21, and cellular SA-β-gal activity (Figs. 1C, 1D, 1E). At a higher concentration, TNFα (20ng/ml) decreased cell proliferation at 3 days and increased senescence markers at 3 and 6 days of treatment (Fig. S1D, E), whereas cells exposed to a lower concentration (5ng/ml) reached senescence at about 26 days of exposure.

Increased intracellular ROS and activation of DNA damage responses are linked mediators of senescence [4, 41, 42]. To delineate the interaction of DNA damage response signals in TNFα-induced senescence, we measured both ROS and DNA damage foci. TNFα-stimulated cells had a robust increase in ROS levels, which subsequently remained elevated (Fig. 1F). Cells exposed to TNFα formed multiple γH2AX foci, a marker of DNA damage, compared to untreated cells (Fig. 1G). Elevated ROS and γH2AX foci were apparent beginning from 24 hours of stimulation and persisted throughout the subsequent period of exposure. The above results demonstrate that TNFα promotes senescence in endothelial cells, accompanied by persistent DNA damage and increased ROS levels.

### Network of inflammatory and SASP gene expression during TNFα-induced senescence

**Figure 2:**
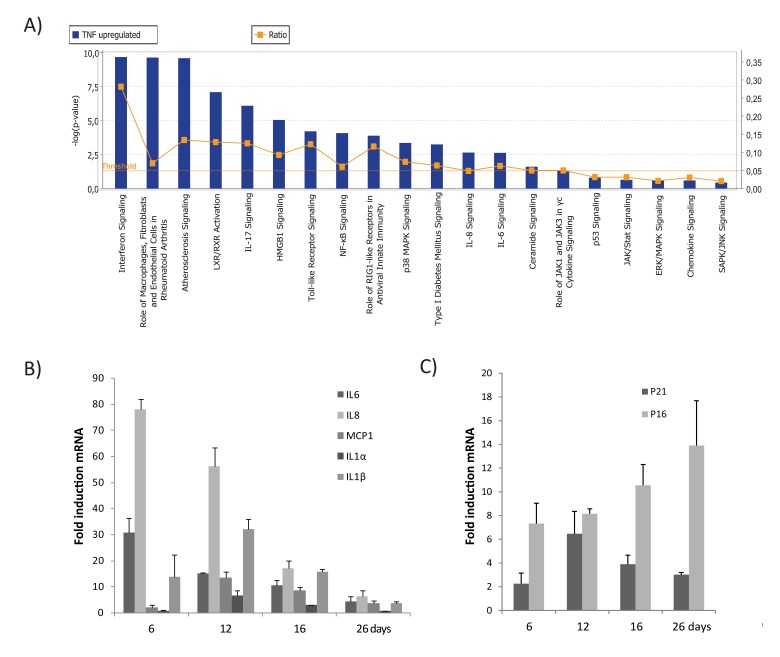
Top canonical pathways identified by IPA analysis in TNFα-induced senescence (**A**) Bar chart represents the top canonical signaling pathways that were influenced during TNFα-induced senescence. p-values were determined using Fisher's exact test with a threshold value of >0.05. Ratios represent the number of genes that mapped to a specific canonical pathway divided by the total number of genes that make up the respective pathway. (**B**) Relative mRNA expression of SASP components in cells treated with TNFα at 6, 16, or 26 days compared to untreated cells. (**C**) Relative mRNA expression of p16 and p21 in cells treated with TNFα (5ng/ml) compared to untreated cells. Results are mean ± standard deviation of n=2 independent experiments.

To explore the molecular background and genes altered during TNFα-induced senescence, we analyzed gene expression profiles of cells made senescent by TNFα *vs*. controls. A total of 238 genes was differentially up-regulated in TNFα-induced senescence compared to control or non-senescent cells. A list of 78 genes up-regulated during TNFα-senescence was selected based on the criteria of adjusted p-value <0.05 and fold change (FC) cut-offs of >1.5. These genes were grouped according to function (Table 1). To identify the biological functions and canonical pathways differentially expressed during TNFα-mediated senes-cence, a gene data set that met the above criteria was exported to the Ingenuity Pathway Analysis (IPA) system. Canonical pathways generated from IPA analysis included NF-kB, p38 MAPK, JAK/STAT, p53, and cytokine-chemokine signaling (Fig 2A). This indicates that molecular crosstalk among diverse signaling pathways occurs during TNFα-induced senescence.

**Table 1:** List of genes differentially up-regulated in TNF-α driven senescence relative to control

Function	Gene Symbol	Description	adj. p.value	Log FC	Fold Change
**Inflammatory genes (Cytokines and Chemokines)**	CXCL6	Chemokine (C-X-C motif) ligand 6 (granulocyte Chemotactic protein 2	4.58E-06	3.7	12.9
	CCL2	Chemokine (C-C motif) ligand 2 (CCL2)	0.002	1.6	3.1
	CCL20	Chemokine (C-C motif) ligand 20 (CCL20)	5.37E-06	3.0	8.1
	CCL5	Chemokine (C-C motif) ligand 5 (CCL5)	0.02	0.9	1.9
	CXCL1	Chemokine (C-X-C motif) ligand 1	3.49E-05	2.2	4.7
	CXCL10	Chemokine (C-X-C motif) ligand 10 (CXCL10)	0.0006	2.2	4.7
	CXCL11	Chemokine (C-X-C motif) ligand 11 (CXCL11)	0.0001	2.6	6.2
	CXCL2	Chemokine (C-X-C motif) ligand 2 (CXCL2)	1.22E-05	2.7	6.3
	CXCL5	Chemokine (C-X-C motif) ligand 5 (CXCL5)	0.02	1.0	2.0
	IL1A	Interleukin 1, alpha (IL1A)	3.10E	2.4	5.1
	IL1B	Interleukin 1, beta (IL1B)	0.02	0.9	1.9
	IL32	Interleukin 32 (IL32)	0.0003	1.8	3.4
	IL4I1	Interleukin 4 induced 1 (IL4I1)	0.01	1.1	2.2
	IL6	Interleukin 6 (interferon, beta 2) (IL6)	0.007	1.1	2.2
	IL8	Interleukin 8 (IL8)	1.22E	2.7	6.4
**Interferon response genes**	MX1	Myxovirus (influenza virus) resistance 1 (MX1)	2.29E-06	4.0	16.1
	IFI44L	Interferon-induced protein 44-like (IFI44L)	1.57E-05	3.2	9.3
	BST2	Bone marrow stromal cell antigen 2 (BST2)	0.0005	2.0	4.1
	DDX58 (RIG-1)	DEAD (Asp-Glu-Ala-Asp) box polypeptide 58 (DDX58)	0.004	1.5	2.9
	DDX60	DEAD (Asp-Glu-Ala-Asp) box polypeptide 60 (DDX60)	0.004	1.7	3.2
	GBP1	Guanylate binding protein 1 (GBP4)	0.005	1.4	2.6
	GBP4	Guanylate binding protein 4 (GBP4)	5.94E	2.7	6.4
	GBP5	Guanylate binding protein 5 (GBP5)	0.01	1.0	2.1
	IFI27	Interferon, alpha-inducible protein 27 (IFI27)	0.0003	1.9	3.7
	IFI30	Interferon, gamma-inducible protein 30 (IFI30)	0.0002	2.1	4.3
	IFI35	Interferon-induced protein 35 (IFI35)	0.001	1.6	3.0
	IFI44	Interferon-induced protein 44 (IFI44)	0.0002	2.0	4.0
	IFI6	Interferon gamma-inducible protein 16 (IFI16)	0.0005	2.0	3.9
	IFIH1	Interferon induced with helicase C domain 1 (IFIH1)	0.0003	2.4	5.2
	IFIT1	Interferon-induced protein with tetratricopeptide repeats 1 (IFIT1)	3.68E-05	3.1	8.7
	IFIT3	Interferon-induced protein with tetratricopeptide repeats 3 (IFIT3)	0.0001	2.4	5.3
	IFIT5	Interferon-induced protein with tetratricopeptide repeats 5 (IFIT5)	0.001	1.5	2.8
	IFITM1	Interferon induced transmembrane protein 1 (IFTM1)	4.72E-05	3.2	9.0
	IRF1	Interferon regulatory factor 1 (IRF1)	0.009	1.2	2.2
	ISG15	ISG15 ubiquitin-like modifier (ISG15)	0.0004	1.9	3.7
	ITGAV	Integrin, alpha V (ITGAV)	0.0003	1.8	3.4
	JAK3	Janus kinase 3 (JAK3)	0.032	0.9	1.8
	MX2	Myxovirus (influenza virus) resistance 2 (MX2)	0.0002	2.6	5.9
	OAS1	2′,5′-oligoadenylate synthetase 1 (OAS1)	0.0002	1.9	3.8
	OAS2	2′-5′-oligoadenylate synthetase 2 (OAS2)	3.04E-05	2.9	7.6
	OAS3	2′-5′-oligoadenylate synthetase 3 (OAS3)	0.001	2.9	7.6
	PSMB9	Proteasome (prosome, macropain) subunit, beta type 9 (large multifunctional peptidase 2) (PSMB9)	1.83E-05	2.7	6.6
	SP110	SP110 nuclear body protein (SP110)	0.006	1.3	2.5
	STAT1	Signal transducer and activator of transcription 1 (STAT1)	0.005	1.2	2.3
	TAP1	Transporter 1, ATP-binding cassette, sub-family B (MDR/TAP) (TAP1)	0.0004	1.8	3.4
	XAF1	XIAP associated factor 1 (XAF1)	2.38E-05	2.6	5.9
**Signal transduction (Receptors, adhesion molecules and immune response genes)**	ATP13A3	ATPase type 13A3 (ATP13A3)	0.0003	1.1	2.1
	CTSS	Cathepsin S (CTSS)	5.37E-06	3.3	9.7
	CD69	CD69 molecule (CD69)	3.95E-09	2.3	5.1
	CD70	CD70 molecule (CD70)	0.005	1.2	2.4
	CLDN1	Claudin 1 (CLDN1)	0.00048	1.7	3.2
	CCND2	Cyclin D2 (CCND2)	0.0037	1.5	2.9
	ICAM1	Intercellular adhesion molecule 1 (ICAM1)	3.49E-05	2.9	7.6
	IL7R	Interleukin 7 receptor (IL7R)	0.0009	1.8	3.4
	IRAK2	Interleukin-1 receptor-associated kinase 2 (IRAK2)	0.001	1.5	2.9
	JAG1	Jagged 1 (Alagille syndrome) (JAG1)	0.0004	1.6	3.0
	JAM2	Junctional adhesion molecule 2 (JAM2)	0.001	1.5	2.8
	JUNB	Jun B proto-oncogene (JUNB)	0.015	1.2	2.3
	MMP1	Matrix metallopeptidase 1 (MMP1)	0.0019	1.7	3.2
	NFKB2	Nuclear factor of kappa light polypeptide gene enhancer in B-cells 2 (p49/p100) (NFKB2)	0.001	1.6	2.9
	NFKBIA	Nuclear factor of kappa light polypeptide gene enhancer in B-cells inhibitor, alpha (NFKBIA)	0.003	1.3	2.5
	NFKBIZ	Nuclear factor of kappa light polypeptide gene enhancer in B-cells inhibitor, zeta (NFKBIZ)	0.001	1.4	2.7
	PTGS2	Prostaglandin-endoperoxide synthase 2 (prostaglandin G/H synthase and cyclooxygenase) (PTGS2)	0.0001	2.3	4.9
	RELB	V-rel reticuloendotheliosis viral oncogene homolog B (RELB)	0.002	1.5	2.9
	SERPINE2	Serpin peptidase inhibitor(nexin, plasminogen activator inhibitor type 1), member 2 (SERPINE 2)	0.045	1.2	2.4
	TGFB2	Transforming growth factor, beta 2 (TGFB2)	0.028	2.8	6.8
	TIFA	TRAF-interacting protein with forkhead-associated domain (TIFA)	0.004	1.6	2.9
	TLR2	Toll-like receptor 2 (TLR2)	0.002	1.3	2.5
	TLR3	Toll-like receptor 3 (TLR3)	0.011	1.3	2.4
	TNFAIP2	Tumor necrosis factor, alpha-induced protein 2 (TNFAIP2)	0.005	1.3	2.5
	TNFAIP3	Tumor necrosis factor, alpha-induced protein 3 (TNFAIP3)	0.0001	2.3	4.9
	TNFRSF11B	Tumor necrosis factor receptor superfamily, member 11b (TNFRSF11B)	0.001	1.7	3.2
	TNFRSF9	Tumor necrosis factor receptor superfamily, member 9 (TNFRSF9)	7.91E-06	3.1	8.5
	TNFSF10	Tumor necrosis factor (ligand) superfamily, member 10 (TNFSF10)	0.005	1.3	2.5
	TNFSF15	Tumor necrosis factor (ligand) superfamily, member 15 (TNFSF15)	0.001	1.7	3.2
	TNIP1	TNFAIP3 interacting protein 1 (TNIP1)	0.005	1.3	2.4
	TNIP3	TNFAIP3 interacting protein 3 (TNIP3)	3.98E-05	2.8	6.8
	IFNGR2	Interferon gamma receptor 2	0.04905	0.8	1.8

Expression of several inflammatory cytokines, chemokines, interleukins, and their receptors was significantly upregulated during TNFα-induced senescence (Table 1), including the cytokines, IL8, IL6, IL1α, IL1β, and IL32 and the chemokines, CXCL1, CXCL2, CXCL5, CXCL6, CXCL10, CXCL11, CCL2, CCL5, and CCL20. Gene products were organized into an interactome network using IPA (Fig. S2). To validate our microarray data, mRNA expression of selected genes (IL6, IL8, IL1α, IL1β, and CCL2/MCP1) and senescence markers (p21 and p16) was assessed by real time PCR (Figs. 2B & 2C). Our findings indicate that senescence established by TNFα leads to an inflammatory signature involving upregulation of genes associated with the secretory pattern of senescent cells.

### Sustained expression of interferon response genes

**Figure 3:**
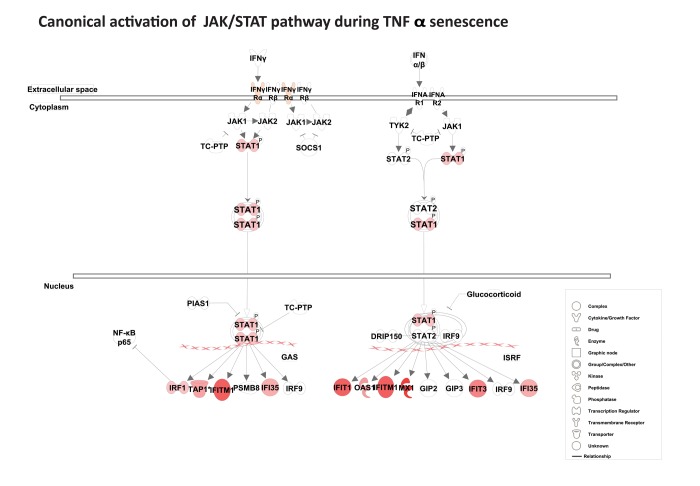
Activation of the canonical JAK/ STAT pathway Pathway analysis using IPA reveals canonical activation of STAT1 acts as a central regulator for JAK/ STAT-mediated interferon response genes during TNFα-induced senescence. The red symbols represent up-regulated genes, with the intensity of node color indicating degree of up-regulation and white indicating genes absent from the list. Shape of nodes denotes functions of gene products.

Among the top scored canonical pathways generated by IPA, the interferon signature was identified as the most prominent gene cluster, involving about 30 of both type I and type II interferon response genes (Table 1). These genes included MX1, ISG15, PSMB9, TAP1, STAT1, three guanylate-binding proteins (GBP1, GBP4, and GBP5), and three 2′5′-oligoadenylate synthetases (OAS1, OAS2, and OAS3). As shown in Fig. S3, the interferon network included several interferon response genes (IFIH1, IFIT5, IFI6, IFI27, IFI30, IFI35, IFI44, IFI44L, DDX58 [RIG-1], and MDA-5) and the p53 target genes, ISG15, and SP110. Several of these genes, including IFI27, IFITM1, IRF1, STAT1, IF16, and TAP1, are known to possess potent anti-proliferative and tumor suppressive properties. Importantly, canonical activation of the JAK/STAT pathway suggests that the transcription factor STAT1 acts as important trans-cription factor for many of the interferon response genes (Fig. 3). Overall, gene expression analysis reveals a strong and sustained interferon signature during TNFα-induced senescence.

### Persistent activation of JAK/STAT signaling in TNFα-induced senescence

**Figure 4:**
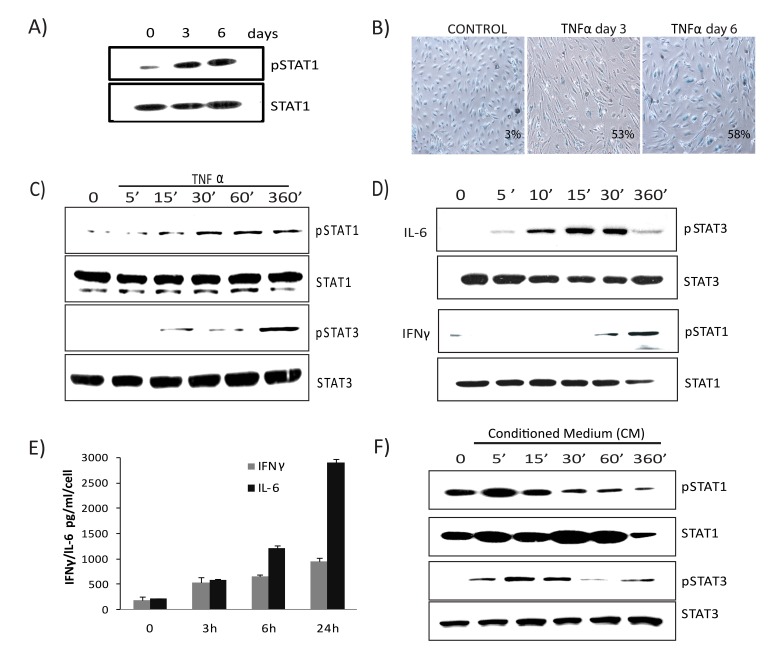
Prolonged activation of JAK/ STAT signaling in TNFα-induced senescence (**A**) Immunoblot detection of p-Ser727-STAT1 and total STAT1 in cells exposed to TNFα 20ng/ml for the indicated times. (**B**) SA-β-gal activity in TNFα (20ng/ml)-treated or control cells for 3 or 6 days. (**C**) Immunoblot detection of p-Ser727-STAT1, total STAT1, p-Tyr705-STAT3, and total STAT3 in cells exposed to TNFα (5ng/ml) for the indicated intervals. (**D**) Immunoblot detection of pSTAT1, total STAT1, pSTAT3, and total STAT3 in cells stimulated with IL6 (10ng/ml) or IFNγ (1ng/ml) for the indicated intervals. (**E**) Secretion of IFNγ/IL6 quantified by ELISA in conditioned medium collected in the presence or absence of TNFα. (**F**) Immunoblot detection of p-Ser727-STAT1, p-Tyr705-STAT3, STAT1, and STAT3 in cells treated with conditioned medium (CM) (cell free-culture supernatants from control and cells stimulated with TNFα for 3 days transferred after 1:4 dilution with fresh culture medium) from TNFα-induced senescent cells or from non-senescent cells for the indicated times.

Gene expression profiling and IPA analysis revealed that the interferon signature and JAK/STAT signaling pathway were preferentially activated and remained sustained during TNFα-mediated senescence. To address the role of the JAK/STAT pathway in TNFα-induced senescence, we examined the long-term kine-tics of STAT activation. The abundance of serine-phosphorylated STAT1 in cells undergoing senescence due to TNFα was sustained at day 3 and remained elevated at day 6, concomitant with the establishment of senescence (Figs. 4A & B). Next we measured early kinetics of STAT1 and STAT3 phosphorylation in response to TNFα. Serine phosphorylation of STAT1 at position 727 in cells exposed to TNFα was detected by 30 minutes, while tyrosine phosphorylation of STAT3 at position 705 was observed at 6 hours (Fig. 4C), indicating a time-dependent phosphorylation pattern. Treatment with IFNγ mediated STAT1 phosphorylation at 30 minutes while IL-6 induced STAT3 phosphory-lation peaked within 10 minutes and later declined (Fig. 4D). These observations suggest that IL-6 induces a direct and transient activation of STAT3 whereas TNFα-mediated STAT3 expression is delayed, indirect, and sustained. As autocrine/paracrine signaling of several cytokines can promote STAT activation, we next analyzed secretion of the major STAT activating ligands, IL6 and IFNγ, in cells senesced by TNFα exposure. Analysis of supernatants collected from cells incubated with TNFα showed a marked increase in secretion of both IL6 and IFNγ compared to control (Fig. 4E). To confirm the involvement of secreted cytokines, we tested the ability of medium conditioned by TNFα to induce STAT activation using supernatants (diluted 1:4 with fresh culture medium) from cultures of control cells or cells stimulated with TNFα for 3 days. Figure 4F shows that the phosphorylated forms of STAT1 and STAT3 peaked early and then slowly decayed in cells treated with conditioned medium compared to control. Unlike the acute and transient response after exposure to either IL6 or conditioned medium, TNFα-induced STAT1/3 activation was chronic and prolonged, coinciding with the development of senescence and the SASP. Furthermore, chronic treatment with recombinant IL6 or conditioned medium containing SASP factors collected from senescent cells failed to induce senescence (Figs. S4A & B), indicating that prolonged activation of the JAK/STAT pathway by TNFα plays a crucial role in stabilizing and retaining the senescent state.

### TNFα initiates a positive feedback loop *via* sustained STAT1/3 activation

**Figure 5:**
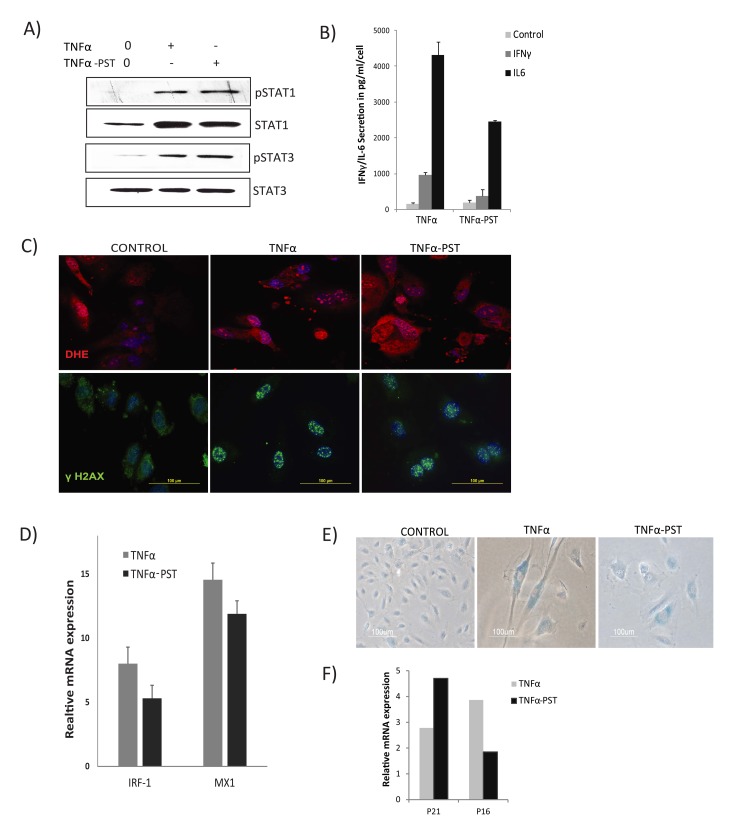
Persistent activation of STAT1/3 Cells were exposed to TNFα (20ng/ml) for 3 days, then washed to remove the residual TNFα and cultured for 3 days in the absence of exogenous TNFα (TNFα-PST). Parallel cultures were exposed to exogenous TNFα throughout the experiment. (**A**) Levels of p-Ser727-STAT1, p-Tyr705-STAT3, and total STAT3 proteins were quantified by immunoblot. (**B**) Secretion of IL-6/IFNγ was assessed in culture supernatants from cells treated with TNFα as indicated. (**C**) Immunodetection of ROS production and γH2AX foci in control or cells treated with TNFα or TNFα-post-stimulated (PST), as indicated. (**D**) Real-time gene expression of IRF1 and MX1 in cells exposed to TNFα or TNFα-PST for 3 days. Results were normalized to internal control TBP and are shown relative to untreated cells. (**E**) SA-β-gal activity in TNFα-treated cells for 3 days or in cells treated with TNFα (20ng/ml) for three days, then washed to remove residual TNFα and left untreated for another 3 days. (F) mRNA expression of p21 and p16 quantified by real-time PCR in cells exposed to TNFα or TNFα-PST for 3 days. Data in D and F represent mean value of ± sd from 2 independent experiments.

The above results suggest that sustained and prolonged activation of JAK/ STAT1/3 signaling could play a crucial role in the TNFα-mediated senescent state. This speculation prompted us to question if prolonged activation of STAT1/3 might be involved in developing a positive feedback loop to sustain the inflammatory network. To address this, we examined the post-stimu-lation effects of TNFα on endothelial cells. Cells were cultured in medium alone or with TNFα for 3 days to induce senescence. Thereafter, cells were washed and cultured in the absence of TNFα for 3 days (post-stimulated, TNFα-PST). Surprisingly, immunoblot analysis showed that phosphorylation of both STAT1 and STAT3 persisted for days in cells senesced by TNFα, even after withdrawal of exogenous TNFα, or in cells that were continuously exposed to TNFα when com-pared to control (Fig. 5A). In contrast, rapid and transient activation of STAT3 was observed in cells treated with IL6 (Fig. 4D), suggesting that primary signals from TNFα specifically contribute to persistent activation of STAT.

To verify the relationship between sustained STAT1/3 activation and development of inflammation and increased ROS levels, we assessed secretion of IL6, IFNγ, and ROS production after withdrawal of exoge-nous TNFα. Supernatants showed increased secretion of both IL6 and IFNγ in TNFα- treated and TNFα withdrawn cells compared to control, indicating that effects of TNFα are sustained and irreversible (Fig. 5B).

Similarly, increased ROS and γH2AX DNA damage foci were found to persist in TNFα-induced senescent cells for days even after removal of the exogenous TNFα (Fig. 5C). These results suggest that prolonged activation of STAT1/3 stimulates a positive auto-regulatory loop *via* an IL6/IFNγ axis, promoting sustained secretion of inflammatory cytokines and ROS production in TNFα-induced senescence.

Although STAT1 is a key mediator of IFN-dependent biological responses, most of the interferon response genes regulated by STAT1 require cooperative action of other transcription factors, such as IRF1 [43]. IRF1 is known to synergize with TNFα signals to drive the production of type I interferons [44]. Considering the possible involvement of STAT1 and IRF1 interactions in the TNFα-induced interferon signature, we examined expression of IRF1 and the interferon response gene MX1. We detected increased and sustained expression of IRF1 and MX1, even 3 days after cessation of TNFα exposure (Fig. 5D), accompanied by sustained expres-sion of senescence markers (Figs. 5E, 5F). These results suggest persistent STAT activation establishes a positive feedback loop promoting a sustained interferon signature and cytokine secretion.

### STAT1/3 activity is required for senescence-associated inflammation and ROS production, while inhibition of STAT modulates TNFα-induced senescence

**Figure 6:**
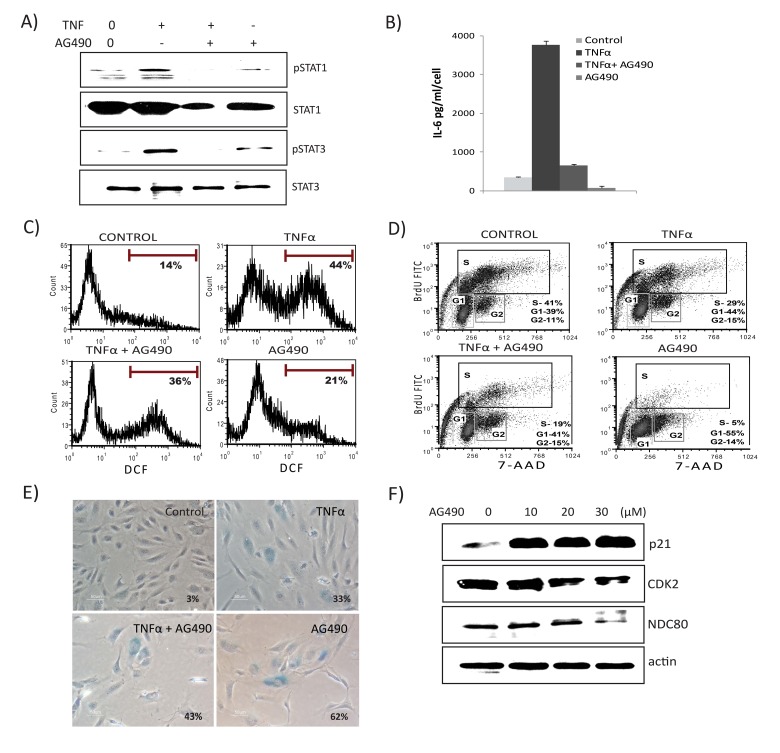
JAK2 inhibitor decreases TNFα-mediated inflammation, ROS levels, and interferon signature Cells were exposed to TNFα alone, TNFα in combination with AG490 (30μM), or AG490 alone for 3 days. (**A**) Immunoblot detection of p-Ser727-STAT1, p-Tyr705-STAT3, and total STAT1 and STAT3. (**B**) Secretion of IL6 was estimated by ELISA in culture supernatants from cells treated with TNFα alone, or in combination with AG490, or AG490 alone for 3 days. (**C**) FACS analysis of ROS levels in cells stimulated either with TNFα, TNFα along with AG490, or AG490 alone for 3 days using DCFDA staining 2′,7′-dichlorofluorescein (DCF) positive cells were analyzed. Inhibition of STAT signals modulates senescence. (**D**) Cell cycle analysis using BrdU and 7- aminoactinomycin D (7-AAD) staning in cells exposed to TNFα, TNFα in combination with AG490, or AG490 alone for 3 days. (**E**) Percentage of SA-β-gal-positive cells. Quantification of SA-β-gal activity in cells stimulated with TNFα 20ng/ml, TNFα in combination with AG490, or AG490 alone for 3 days. The data represent means of 3 independent counts of 200 cells from 2 independent experiments. (**F**) Effect of AG490 on cell cycle regulatory proteins. Western analysis performed using cells treated with AG490 for 3 days and blotted against anti-p21, CDK2, and NDC80. Actin serves as loading control.

To explore the functional role of the JAK/STAT pathway in TNFα-mediated senescence, we inhibited JAK2 receptor kinase using the inhibitor AG-490. As shown in Fig. 6A, pretreatment with AG-490 blunted TNFα-induced activation of both STAT1 and STAT3. Importantly, inhibition of JAK markedly decreased TNFα-induced secretion of both IL6 and ROS (Figs. 6B & 6C), indicating a requirement for STAT1/3 activity in TNFα-mediated inflammatory and DNA damage responses. Conversely, treatment with AG490 alone decreased the percentage of BrdU-incorporating cells, inhibited S phase entry, and resulted in cell cycle arrest (Fig. 6D). The presence of both TNFα and AG-490 decreased proliferation and increased SA-β-gal-positive cells compared to TNFα alone (Fig. 6E). Since the STAT pathway is involved in a wide range of cellular processes including proliferation, apoptosis, and differentiation, we tested if cells treated with AG-490 undergo senescence. Surprisingly, inhibition of STAT1/3 by AG-490 increased expression of senes-cence markers, including SA-β-gal and p21, whereas expression of cell cycle regulatory genes, such as CDK2 and NDC80, were decreased (Fig. 6F). Similar results were seen using the STAT3 inhibitor curcubitacin (data not shown). As p38MAPK (p38) activation plays a significant role in TNFα signaling and senescence, we also examined the role of p38. Chemical inhibition of p38 activation using SB203580 (SB) rescued cells not only from senescence, but also diminished TNFα-induced IL-6 secretion and ROS (Figs. S5A, B, & C). Furthermore, inhibition of p38 activation attenuated phosphorylation of STAT1, indicating that p38 acts as an upstream regulator of STAT1 and thus its activation requires p38 signals (Fig. S5D). These results indicate that a STAT1/3 pathway plays a dual in role in modulating TNFα-mediated senescence. On the one hand, this pathway is required for promoting TNFα-induced inflammatory and damage responses. On the other, interfering with STAT signaling itself accelerates senescence.

## DISCUSSION

The biology of cellular senescence is complex, involving cascades of regulatory events. Mounting evidence indicates cytokine secretion and expression can promote and reinforce senescence through both paracrine and autocrine mechanisms [17, 19, 45, 46]. Pro-inflammatory signals and chronic inflammation cause growth arrest, DNA damage, and are often integral components of senescence both in normal and cancer cells [6, 33, 37, 47–49]. Gene expression changes during inflammatory senescence and pathways linking inflammation to senescence still need to be elucidated. Here, we established an experimental model of senes-cence triggered by the pro-inflammatory cytokine, TNFα. We mapped pathways linking inflammation to senescence and showed that TNFα operates a positive feedback mechanism through JAK/STAT signaling. Our study provides a comprehensive overview of gene expression changes, novel transcriptional pathways, and their putative targets involved in cytokine-induced senescence. Our gene expression data suggest that TNFα-induced senescence represents a novel mecha-nism and shares some common features with the more typical senescence phenotype. Genes up-regulated by TNFα, including ICAM-1, MMP1, ATP13A3, RIG1, PTGS2, and MCP1, have been shown to be elevated in senescent cells [50–54]. Furthermore, many TNFα-induced secreted factors, including IL8, IL6, IL1α, and IL1β, are important SASP components known to reinforce the senescent phenotype [9, 17, 20–22, 50]. Our data provide molecular support for the notion that multiple signaling cascades, including NF-κB, TLRs, JAK/STAT, and MAPK, integrate to drive the complex senescence phenotype. During development of senes-cence, several pathways control initiation and maintenance of the SASP [20, 21, 28, 30, 33, 46, 48]. We hypothesize that, in addition to positive feed-forward mechanisms, negative feedback loops also contribute to restrain the SASP network during senescence. We observed that secretion of IL6 and expression of other SASP factors, including ICAM and VCAM, were elevated during the initial phase of TNFα-induced senescence. These factors declined after senescence had become established (Fig. 2B & Figs. S6A, B, & C). We speculate that during the onset of senescence, a robust inflammatory response amplifies a series of downstream signaling cascades. The low-level and sustained secretion of the SASP factors during late-phase of senescence may limit deleterious effects, while allowing senescent growth arrest to be maintained. Feedback loops mediated by miRNAs are also involved in controlling senescence-associated inflammation: up-regulation of miR-146a/b in senescent fibroblasts negatively modulates secretion of inflammatory molecules such as IL6 and IL8 [55]. Consistent with this possibility, our results show that expression of miR-146a is up-regulated during TNFα-mediated senescence (unpublished data). Further investigation is needed to dissect the regulatory role of miRNA loops in TNFα-mediated senescence.

In this study, we used human endothelial cells as a model to study senescence because endothelial cells are active participants in inflammatory responses. Endo-thelial dysfunction occurring after chronic injury and inflammation is associated with development and progression of many age-related diseases [56, 57]. Inflammation is associated with persistent DNA damage and increased ROS production during senescence [6, 26, 51]. Our results are consistent with several studies indicating that cytokine signals, persistent DDR, and ROS production are interconnected through a feedback loop to reinforce senescence both in cell culture and *in vivo* models [6, 30, 46, 58]. However, neither the mechanism of the TNFα-dependent interferon response, nor the positive feedback mechanism operated through sustained STAT activation connecting cytokine loops and ROS, have been addressed in these previous studies.

Although multiple pathways impinge upon TNFα-mediated senescence, we show that the JAK/STAT pathway is a key mediator of inflammatory and interferon signatures. We previously reported that inhibiting the JAK/STAT pathway suppresses the SASP in preadipocytes and endothelial cells, together with decreased adipose tissue inflammation [34]. Likewise, studies have shown that the combination of the T-helper cell cytokines, IFNγ and TNFα, or genotoxic drugs induces tumor cell senescence and involves the JAK/STAT pathway [30, 33, 47]. This suggests that the senescence-inducing pattern involving STATs is not limited to HUVECs alone. The regulatory role of STAT1/3 signals in TNFα-mediated senescence is suggested by our findings that: i) prolonged activation of STAT1/3 initiated by TNFα acts as a positive regulator of transcription of many interferon response genes, ii) levels of upstream mediators of STAT1 and STAT3, such as IL6 and IFNγ, are increased, and iii) TNFα, in synergy with IFN-γ, activates a positive autocrine to loop to sustain interferon response genes and inflammation, in line with previous reports [44, 59–61]. Together, these findings suggest that persistently activated STAT1/3, together with inflammatory and DNA damage signals, acts as a central regulator in promoting and perpetuating multiple feedback loops in senescence mediated by TNFα.

**Figure 7:**
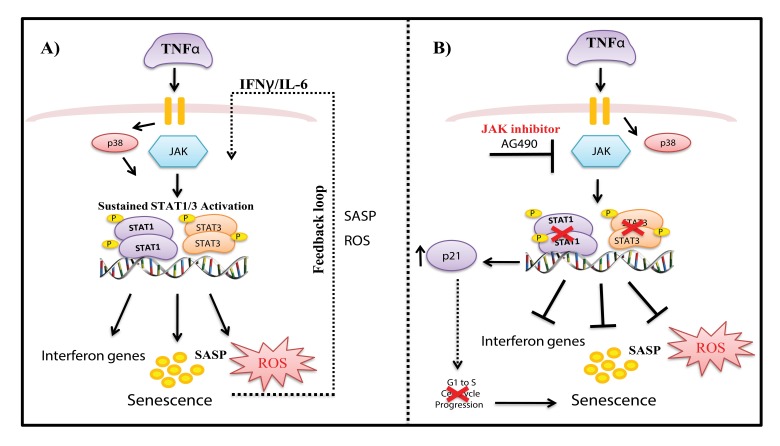
Model of mechanisms involved in TNFα-induced senescence of HUVECs (**A**) TNFα activates JAK/STAT and p38 signaling pathways, which mediate increased expression of STAT1/3 phosphorylation. Activation of the JAK pathway leads to persistent phosphorylation of STAT1/3 signaling, which together with ROS, interferon genes, and other SASP components, drives a positive auto-regulatory loop, leading to sustained inflammation and stable senescence. (**B**) Inhibition of STAT1/3 with the JAK inhibitor AG490 decreased ROS and IL-6 production and decreased expression of interferon response genes. On the other hand, blockade of STAT1/3 expression decreased S phase entry of cells and increased p21 expression, leading to senescence.

STATs play major role in expression of genes that control cell cycle progression, differentiation, pro-liferation, and apoptosis and constitutively active STATs have been associated with a range of human cancers [62–64]. Despite the fact that activated STATs are highly linked to proliferation, we show here their contribution to senescence and inflammation, support-ing the dual role in proliferation and tumor suppression [33, 34, 63, 65]. A model of our findings is depicted in Fig. 7. TNFα-induced senescence initiates a positive auto-regulatory loop *via* a persistently activated STAT-pathway leading to sustained DNA damage and cytokine secretion, thereby maintaining and retaining the senescent state. Unexpectedly, inhibition of STAT1/3 with AG490 did not rescue cells from TNFα-induced senescence, but rather suppressed genes involved in interferon gene expression, inflammation, and ROS production. AG490 treatment increased p21 expression and repressed cell cycle progression genes (*e.g*., CDK2, NDC80), leading to senescence. Further studies would be interesting to clarify the role and mechanisms involved in AG490- induced senescence, as it may be a potential therapeutic target. Inhibition of p38 reduced phosphorylation of STAT, decreased IL-6 expression and ROS production, and rescued cells from senescence. As noted in previous reports [27, 28, 48, 66, 67], we show that stress-induced activation of STAT1 at Ser727 requires p38 activity. Consistent with previous findings we also show that the p38 MAPK pathway plays an important role in regulating senescence.

Our study suggests the JAK/STAT1/3 pathway plays a dual modulatory role during cytokine induced senescence in endothelial cells. On the one hand, STAT1/3 mediates TNFα-induced senescence by promoting expression of anti-proliferative, inflamma-tory, and tumor suppressor genes involved in main-taining the senescent state. On the other, inhibition of JAK/STAT1/3 activation triggers an unconventional type of senescence that does not involve cytokine or ROS production. Similarly, cells that senesce by over-expressing cell cycle regulators such as p16 lack DNA damage and do not initiate a cytokine response [68]. Considering its key role, the JAK/ STAT pathway could be a therapeutic target for treating senescence-related inflammatory diseases. In conclusion, our results suggest that pathways connecting inflammation to senescence are complex, involving multiple regulatory circuits and self-reinforcing inflammatory feedback loops.

## MATERIALS AND METHODS

### Reagents and antibodies

Recombinant human TNFα, IL6, and IFNγ were purchased from Bionova (Madrid, Spain), AG490 from Calbiochem (Darmstadt, Germany), antibodies against pSTAT1^Ser727^, pSTAT3^Ser705^, STAT1, STAT3, and p-p38 from Cell Signaling (Danvers, MA, USA), monoclonal antibodies against p21 and p16 from Thermo Scientific (Fremont, CA, USA), γH2AX from Millipore (Billerica, MA, USA), anti-CDK2 from Abcam (Cambridge, MA, USA), anti-NDC80 from BD Biosciences (San Diego, CA, USA), anti-actin, and anti-β-tubulin from Sigma (St Louis, MS, USA), and anti-human CD54 (ICAM-1) and anti-human CD106 (VCAM) from BD Biosciences.

### Cell culture

Human umbilical vein endothelial cells (HUVECs) purchased from Clonetics/Lonza (Basel, Switzerland) were grown in EGM-2 medium supplemented with growth factors (EGM-2 bullet kit; Clonetics/Lonza). Monolayer HUVECs were maintained in 25cm^2^ or 75cm^2^ tissue culture flasks or 100mm tissue culture plates at 37^°^C in a humidified incubator with 5% CO_2_ and 95% air.

### Induction of senescence and long-term growth curves

To induce TNFα-mediated senescence, HUVECs were continuously cultured in the presence or absence of recombinant human TNFα for the indicated periods. Culture medium was changed every other day with new TNFα. Stimulation with TNFα resulted in dose- and time-dependent induction of senescence in endothelial cells. With increasing concentration, TNFα at (20ng/ml) triggered senescence by 3 days of treatment, whereas cells treated with 5ng/ml of TNFα entered senescence at about 26 days (Fig. 1A). Growth curves were plotted by determining cell numbers at the end of each passage in TNFα-treated and -untreated cells. Population doublings (PD) were calculated using the formula PD = (lg [number of cells harvested] – lg [number of cells seeded]) / lg2 [69]. Population doubling time (PDT) was obtained by the formula PDT = tlg2/ (lg [number of cells harvested] – lg [number of cells seeded]), where t is the time of the culture (in hours). In all experiments, early passage HUVECs were used to start (p2-p3).

### Experimental design for microarray analysis

To determine the long-term and sustained gene expression changes in senescence driven by TNFα, we compared expression profiles of cells chronically cultured in the presence or absence of 5ng/ml TNFα for 26 days. For the TNFα-treated group, we chose day 26 as the relevant time point, since population doubling times and morphological changes indicated the cells had achieved permanent growth arrest or were senescent. Three independently cultured samples for each experi-mental group of cells made senescent by TNFα were compared with corresponding passaged cells from the untreated control group.

### RNA isolation and gene expression analysis

Total RNA was extracted from sub-confluent cultures from the above-mentioned experimental groups using tri-reagent and stored at -80^°^C until processed. Changes in gene transcription were analyzed by hybridization to Affymetrix arrays. Three biological replicates from each experimental time point were analyzed using the Affymetrix Human Gene Chip® Gene 1.0 ST Array System. The Human Gene Chip® Gene 1.0 ST array interrogates 28,869 well-annotated genes with 764,885 distinct probe sets. The microarray data were deposited to the GEO database (accession number GSE98081).

### Data analysis, identification of networks and canonical pathways

Array data were normalized and summarized with RMA software. Gene filtering, pair-wise comparisons, t-tests, and PCA were obtained using Bio-conductor packages http://www.bioconductor.org [70] based on the R package http://www.rproject.org [71]. Genes that were differentially expressed by 1.5-fold or more and with an adjusted P value of p<0.05 were analyzed. Data lists of all the genes that satisfied these criteria were analyzed by Ingenuity Pathway Analysis (IPA) software (Ingenuity^®^ Systems, Redwood City, CA, USA) http://www.ingenuity.com. In order to identify func-tional modules involved in responses to the compared stimuli, genome-wide interactions, networks, and canonical pathways were generated using IPA.

### BrdU incorporation

BrdU incorporation was measured using a BrdU Flow Kit (BD Biosciences) following the manufacturer's protocol with modifications. BrdU was added to culture medium at a final concentration of 10μM. Six hours after addition of BrdU, cells were harvested, washed once with 1x phosphate-buffered saline, and fixed in 100μl of Cytofix/Cytoperm for 30 minutes at room temperature. After DNase treatment, BrdU-incorporated cells were then stained with FITC-conjugated anti-BrdU antibody and 7-aminoactinomycin D. Cell cycle profiles were analyzed using a FACS caliber flow cyto-meter (BD Biosciences). Data analysis was performed using FCS express software.

### Senescence-associated β-galactosidase assay (SA-β-gal)

SA-β-gal activity was detected as described previously [11] using a senescence staining kit according to the manufacturer's recommendations (Sigma). Stained cells were then visualized and photographed with a Nikon microscope. The percentage of cells positive for SA-β-gal was determined in duplicate in 200 cells per sample.

### Cytokine secretion

Supernatants from cell cultures were collected on different days of incubation and stored at -80°C until used. Concentrations of IL6 and IFN-gamma (IFNγ) secreted in supernatants were quantified using a comer-cial ELISA kit according to the manufacturer's instruct-tions (OptEIA; BD Biosciences, San Diego CA, USA).

### Western blots

Western blot analysis was performed by eluting whole cell-lysates using 2x Laemmeli sample buffer followed by sonication of samples for 10 seconds. Samples were resolved in SDS-PAGE. Proteins were then transferred onto nitrocellulose membranes. Blocked membranes were incubated with the indicated antibodies. Immuno-reactive bands were developed using supersignal Western substrate (Immobilon, Millipore) and visualized on AGFA (CP-BU) films following auto-mated development.

### ROS production

Cells were stimulated with TNFα or medium alone for the indicated times and labeled with 10μM of 2′,7′-dichlorodihydrofluorescein diacetate (H2DCFDA; Molecular Probes, Carlsbad, CA, USA) for 30 minutes at 37°C. Intracellular ROS levels in labeled cells were assessed using a Becton Dickinson FACS Caliber flow scan.

### Indirect immunofluorescence

For indirect immunofluorescence, cells grown on coverslips were fixed with 3% formaldehyde in PBS at room temperature for 10 minutes, blocked with 10% fetal bovine serum in PBS for 30 minutes, and permeabilized with 0.2% Triton X-100, 3% BSA in PBS for 30 minutes. Cells were then incubated with primary antibody against anti-phospho-gamma H2AX (Millipore) for 1 hour and stained with Alexa Fluor 488- (DAKO, Carpinteria, CA, USA) conjugated secondary antibody for 30 minutes. DNA was counter-stained with DAPI to visualize nuclei.

### Real-time PCR

cDNA was synthesized using a high capacity cDNA reverse transcription kit from Applied Biosystems. Quantitative real time PCR reactions were performed with 1x Taqman universal PCR master mix (Applied Biosystems) in a MicroAmp optical 96-well reaction plate. All reactions were done in triplicate using two dilutions (1/500, 1/1000) each in 20μl reaction mix consisting of 9μl of cDNA, 1μM of each primer, and 10μl Taqman mastermix. The plates were then covered tightly with an optical adhesive cover and centrifuged at 1400 x g for 30 seconds at 4°C. PCR reactions were run on an ABI Prism 7500 (Applied Biosystems) and results were analyzed using the same software. Probes and primers were purchased from Life Technologies. Relative gene expression levels were normalized to GAPDH/TBP.

## SUPPLEMENTARY MATERIAL FIGURES



## References

[1] Tchkonia T, Zhu Y, van Deursen J, Campisi J, Kirkland JL (2013). Cellular senescence and the senescent secretory phenotype: therapeutic opportunities. J Clin Invest.

[2] Hayflick L, Moorhead PS (1961). The serial cultivation of human diploid cell strains. Exp Cell Res.

[3] Sahin E, Colla S, Liesa M, Moslehi J, Müller FL, Guo M, Cooper M, Kotton D, Fabian AJ, Walkey C, Maser RS, Tonon G, Foerster F (2011). Telomere dysfunction induces metabolic and mitochondrial compromise. Nature.

[4] von Zglinicki T, Saretzki G, Ladhoff J, d'Adda di Fagagna F, Jackson SP (2005). Human cell senescence as a DNA damage response. Mech Ageing Dev.

[5] Herbig U, Jobling WA, Chen BP, Chen DJ, Sedivy JM (2004). Telomere shortening triggers senescence of human cells through a pathway involving ATM, p53, and p21(CIP1), but not p16(INK4a). Mol Cell.

[6] Correia-Melo C, Hewitt G, Passos JF (2014). Telomeres, oxidative stress and inflammatory factors: partners in cellular senescence?. Longev Healthspan.

[7] Dumont P, Chainiaux F, Eliaers F, Petropoulou C, Remacle J, Koch-Brandt C, Gonos ES, Toussaint O (2002). Overexpression of apolipoprotein J in human fibroblasts protects against cytotoxicity and premature senescence induced by ethanol and tert-butylhydroperoxide. Cell Stress Chaperones.

[8] Toussaint O, Dumont P, Remacle J, Dierick JF, Pascal T, Frippiat C, Magalhaes JP, Zdanov S, Chainiaux F (2002). Stress-induced premature senescence or stress-induced senescence-like phenotype: one in vivo reality, two possible definitions?. Sci World J.

[9] Campisi J, d'Adda di Fagagna F (2007). Cellular senescence: when bad things happen to good cells. Nat Rev Mol Cell Biol.

[10] Campisi J (2005). Senescent cells, tumor suppression, and organismal aging: good citizens, bad neighbors. Cell.

[11] Dimri GP, Lee X, Basile G, Acosta M, Scott G, Roskelley C, Medrano EE, Linskens M, Rubelj I, Pereira-Smith O (1995). A biomarker that identifies senescent human cells in culture and in aging skin in vivo. Proc Natl Acad Sci USA.

[12] Gire V, Roux P, Wynford-Thomas D, Brondello JM, Dulic V (2004). DNA damage checkpoint kinase Chk2 triggers replicative senescence. EMBO J.

[13] Zhang R, Chen W, Adams PD (2007). Molecular dissection of formation of senescence-associated heterochromatin foci. Mol Cell Biol.

[14] Narita M, Nũnez S, Heard E, Narita M, Lin AW, Hearn SA, Spector DL, Hannon GJ, Lowe SW (2003). Rb-mediated heterochromatin formation and silencing of E2F target genes during cellular senescence. Cell.

[15] Freund A, Orjalo AV, Desprez PY, Campisi J (2010). Inflammatory networks during cellular senescence: causes and consequences. Trends Mol Med.

[16] Coppé JP, Patil CK, Rodier F, Sun Y, Muñoz DP, Goldstein J, Nelson PS, Desprez PY, Campisi J (2008). Senescence-associated secretory phenotypes reveal cell-nonautonomous functions of oncogenic RAS and the p53 tumor suppressor. PLoS Biol.

[17] Kuilman T, Michaloglou C, Vredeveld LC, Douma S, van Doorn R, Desmet CJ, Aarden LA, Mooi WJ, Peeper DS (2008). Oncogene-induced senescence relayed by an interleukin-dependent inflammatory network. Cell.

[18] Kuilman T, Peeper DS (2009). Senescence-messaging secretome: SMS-ing cellular stress. Nat Rev Cancer.

[19] Acosta JC, O'Loghlen A, Banito A, Guijarro MV, Augert. A, Raguz S, Fumagalli M, Da Costa M, Brown C, Popov N, Takatsu Y, Melamed J, d'Adda di Fagagna F (2008). Chemokine signaling via the CXCR2 receptor reinforces senescence. Cell.

[20] Acosta JC, Banito A, Wuestefeld T, Georgilis A, Janich P, Morton JP, Athineos D, Kang TW, Lasitschka F, Andrulis M, Pascual G, Morris KJ, Khan S (2013). A complex secretory program orchestrated by the inflammasome controls paracrine senescence. Nat Cell Biol.

[21] Hubackova S, Krejcikova K, Bartek J, Hodny Z (2012). IL1- and TGFβ-Nox4 signaling, oxidative stress and DNA damage response are shared features of replicative, oncogene-induced, and drug-induced paracrine ‘bystander senescence'. Aging (Albany NY).

[22] Orjalo AV, Bhaumik D, Gengler BK, Scott GK, Campisi J (2009). Cell surface-bound IL-1alpha is an upstream regulator of the senescence-associated IL-6/IL-8 cytokine network. Proc Natl Acad Sci USA.

[23] Fumagalli M, d'Adda di Fagagna F (2009). SASPense and DDRama in cancer and ageing. Nat Cell Biol.

[24] Rodier F, Campisi J, Bhaumik D (2007). Two faces of p53: aging and tumor suppression. Nucleic Acids Res.

[25] Fumagalli M, Rossiello F, Mondello C, d'Adda di Fagagna F (2014). Stable cellular senescence is associated with persistent DDR activation. PLoS One.

[26] Ren JL, Pan JS, Lu YP, Sun P, Han J (2009). Inflammatory signaling and cellular senescence. Cell Signal.

[27] Zhang Y, Herbert BS, Rajashekhar G, Ingram DA, Yoder MC, Clauss M, Rehman J (2009). Premature senescence of highly proliferative endothelial progenitor cells is induced by tumor necrosis factor-alpha via the p38 mitogen-activated protein kinase pathway. FASEB J.

[28] Freund A, Patil CK, Campisi J (2011). p38MAPK is a novel DNA damage response-independent regulator of the senescence-associated secretory phenotype. EMBO J.

[29] Zhou H, Huang B, Han Y, Jin R, Chen S (2013). Probucol inhibits JAK2-STAT pathway activation and protects human glomerular mesangial cells from tert-butyl hydroperoxide induced premature senescence. Can J Physiol Pharmacol.

[30] Novakova Z, Hubackova S, Kosar M, Janderova-Rossmeislova L, Dobrovolna J, Vasicova P, Vancurova M, Horejsi Z, Hozak P, Bartek J, Hodny Z (2010). Cytokine expression and signaling in drug-induced cellular senescence. Oncogene.

[31] Adler AS, Kawahara TL, Segal E, Chang HY (2008). Reversal of aging by NFkappaB blockade. Cell Cycle.

[32] Hayden MS, Ghosh S (2008). Shared principles in NF-kappaB signaling. Cell.

[33] Hubackova S, Kucerova A, Michlits G, Kyjacova L, Reinis M, Korolov O, Bartek J, Hodny Z (2016). induces oxidative stress, DNA damage and tumor cell senescence via TGFβ/SMAD signaling-dependent induction of Nox4 and suppression of ANT2. Oncogene.

[34] Xu M, Tchkonia T, Ding H, Ogrodnik M, Lubbers ER, Pirtskhalava T, White TA, Johnson KO, Stout MB, Mezera V, Giorgadze N, Jensen MD, LeBrasseur NK, Kirkland JL (2015). JAK inhibition alleviates the cellular senescence-associated secretory phenotype and frailty in old age. Proc Natl Acad Sci USA.

[35] Franceschi C, Bonafè M, Valensin S, Olivieri F, De Luca M, Ottaviani E, De Benedictis G (2000). Inflamm-aging. An evolutionary perspective on immunosenescence. Ann N Y Acad Sci.

[36] Xu M, Palmer AK, Ding H, Weivoda MM, Pirtskhalava T, White TA, Sepe A, Johnson KO, Stout MB, Giorgadze N, Jensen MD, LeBrasseur NK, Tchkonia T, Kirkland JL (2015). Targeting senescent cells enhances adipogenesis and metabolic function in old age. eLife.

[37] Jurk D, Wilson C, Passos JF, Oakley F, Correia-Melo C, Greaves L, Saretzki G, Fox C, Lawless C, Anderson R, Hewitt G, Pender SL, Fullard N (2014). Chronic inflammation induces telomere dysfunction and accelerates ageing in mice. Nat Commun.

[38] Zhu Y, Armstrong JL, Tchkonia T, Kirkland JL (2014). Cellular senescence and the senescent secretory phenotype in age-related chronic diseases. Curr Opin Clin Nutr Metab Care.

[39] Aggarwal BB (2003). Signalling pathways of the TNF superfamily: a double-edged sword. Nat Rev Immunol.

[40] Holtmann MH, Neurath MF (2004). Differential TNF-signaling in chronic inflammatory disorders. Curr Mol Med.

[41] Rodier F, Coppé JP, Patil CK, Hoeijmakers WA, Muñoz DP, Raza SR, Freund A, Campeau E, Davalos AR, Campisi J (2009). Persistent DNA damage signalling triggers senescence-associated inflammatory cytokine secretion. Nat Cell Biol.

[42] Correia-Melo C, Marques FD, Anderson R, Hewitt G, Hewitt R, Cole J, Carroll BM, Miwa S, Birch J, Merz A, Rushton MD, Charles M, Jurk D (2016). Mitochondria are required for pro-ageing features of the senescent phenotype. EMBO J.

[43] Ramsauer K, Sadzak I, Porras A, Pilz A, Nebreda AR, Decker T, Kovarik P (2002). p38 MAPK enhances STAT1-dependent transcription independently of Ser-727 phosphorylation. Proc Natl Acad Sci USA.

[44] Yarilina A, Park-Min KH, Antoniv T, Hu X, Ivashkiv LB (2008). TNF activates an IRF1-dependent autocrine loop leading to sustained expression of chemokines and STAT1-dependent type I interferon-response genes. Nat Immunol.

[45] Bartek J, Hodny Z, Lukas J (2008). Cytokine loops driving senescence. Nat Cell Biol.

[46] Passos JF, Nelson G, Wang C, Richter T, Simillion C, Proctor CJ, Miwa S, Olijslagers S, Hallinan J, Wipat A, Saretzki G, Rudolph KL, Kirkwood TB, von Zglinicki T (2010). Feedback between p21 and reactive oxygen production is necessary for cell senescence. Mol Syst Biol.

[47] Braumüller H, Wieder T, Brenner E, Aßmann S, Hahn M, Alkhaled M, Schilbach K, Essmann F, Kneilling M, Griessinger C, Ranta F, Ullrich S, Mocikat R (2013). T-helper-1-cell cytokines drive cancer into senescence. Nature.

[48] Kim HD, Yu SJ, Kim HS, Kim YJ, Choe JM, Park YG, Kim J, Sohn J (2013). Interleukin-4 induces senescence in human renal carcinoma cell lines through STAT6 and p38 MAPK. J Biol Chem.

[49] Kim KS, Kang KW, Seu YB, Baek SH, Kim JR (2009). Interferon-gamma induces cellular senescence through p53-dependent DNA damage signaling in human endothelial cells. Mech Ageing Dev.

[50] Davalos AR, Coppe JP, Campisi J, Desprez PY (2010). Senescent cells as a source of inflammatory factors for tumor progression. Cancer Metastasis Rev.

[51] Freund A, Orjalo AV, Desprez PY, Campisi J (2010). Inflammatory networks during cellular senescence: causes and consequences. Trends Mol Med.

[52] Jin HJ, Lee HJ, Heo J, Lim J, Kim M, Kim MK, Nam HY, Hong GH, Cho YS, Choi SJ, Kim IG, Shin DM, Kim SW (2016). Senescence-Associated MCP-1 Secretion Is Dependent on a Decline in BMI1 in Human Mesenchymal Stromal Cells. Antioxid Redox Signal.

[53] Liu F, Wu S, Ren H, Gu J (2011). Klotho suppresses RIG-I-mediated senescence-associated inflammation. Nat Cell Biol.

[54] Shelton DN, Chang E, Whittier PS, Choi D, Funk WD (1999). Microarray analysis of replicative senescence. Curr Biol.

[55] Bhaumik D, Scott GK, Schokrpur S, Patil CK, Orjalo AV, Rodier F, Lithgow GJ, Campisi J (2009). MicroRNAs miR-146a/b negatively modulate the senescence-associated inflammatory mediators IL-6 and IL-8. Aging (Albany NY).

[56] Chen J, Goligorsky MS (2006). Premature senescence of endothelial cells: methusaleh's dilemma. Am J Physiol Heart Circ Physiol.

[57] Minamino T, Komuro I (2007). Vascular cell senescence: contribution to atherosclerosis. Circ Res.

[58] Nelson G, Wordsworth J, Wang C, Jurk D, Lawless C, Martin-Ruiz C, von Zglinicki T (2012). A senescent cell bystander effect: senescence-induced senescence. Aging Cell.

[59] Xu G, Zhang Y, Zhang L, Roberts AI, Shi Y (2009). C/EBPbeta mediates synergistic upregulation of gene expression by interferon-gamma and tumor necrosis factor-alpha in bone marrow-derived mesenchymal stem cells. Stem Cells.

[60] Sekine N, Ishikawa T, Okazaki T, Hayashi M, Wollheim CB, Fujita T (2000). Synergistic activation of NF-kappab and inducible isoform of nitric oxide synthase induction by interferon-gamma and tumor necrosis factor-alpha in INS-1 cells. J Cell Physiol.

[61] Lee AH, Hong JH, Seo YS (2000). Tumour necrosis factor-alpha and interferon-gamma synergistically activate the RANTES promoter through nuclear factor kappaB and interferon regulatory factor 1 (IRF-1) transcription factors. Biochem J.

[62] Calò V, Migliavacca M, Bazan V, Macaluso M, Buscemi M, Gebbia N, Russo A (2003). STAT proteins: from normal control of cellular events to tumorigenesis. J Cell Physiol.

[63] Thomas SJ, Snowden JA, Zeidler MP, Danson SJ (2015). The role of JAK/STAT signalling in the pathogenesis, prognosis and treatment of solid tumours. Br J Cancer.

[64] Yu H, Pardoll D, Jove R (2009). STATs in cancer inflammation and immunity: a leading role for STAT3. Nat Rev Cancer.

[65] Kojima H, Inoue T, Kunimoto H, Nakajima K (2013). IL-6-STAT3 signaling and premature senescence. JAK-STAT.

[66] Goh KC, Haque SJ, Williams BR (1999). p38 MAP kinase is required for STAT1 serine phosphorylation and transcriptional activation induced by interferons. EMBO J.

[67] Kovarik P, Stoiber D, Eyers PA, Menghini R, Neininger A, Gaestel M, Cohen P, Decker T (1999). Stress-induced phosphorylation of STAT1 at Ser727 requires p38 mitogen-activated protein kinase whereas IFN-gamma uses a different signaling pathway. Proc Natl Acad Sci USA.

[68] Coppé JP, Rodier F, Patil CK, Freund A, Desprez PY, Campisi J (2011). Tumor suppressor and aging biomarker p16(INK4a) induces cellular senescence without the associated inflammatory secretory phenotype. J Biol Chem.

[69] Kurz DJ, Decary S, Hong Y, Trivier E, Akhmedov A, Erusalimsky JD (2004). Chronic oxidative stress compromises telomere integrity and accelerates the onset of senescence in human endothelial cells. J Cell Sci.

[70] Gentleman RC, Carey VJ, Bates DM, Bolstad B, Dettling M, Dudoit S, Ellis B, Gautier L, Ge Y, Gentry J, Hornik K, Hothorn T, Huber W (2004). Bioconductor: open software development for computational biology and bioinformatics. Genome Biol.

[71] Kennedy RE, Kerns RT, Kong X, Archer KJ, Miles MF (2006). SScore: an R package for detecting differential gene expression without gene expression summaries. Bioinformatics.

