# Supplementary material for: TNFα-senescence initiates a STAT-dependent positive feedback loop, leading to a sustained interferon signature, DNA damage, and cytokine secretion
Source: Aging (Albany NY). 2017 Nov 22;9(11):2411–35. doi: 10.18632/aging.101328 (PMC5723694; doi:10.18632/aging.101328)
Supplement: Supplementary file 1 [file aging-09-2411-s001.pdf]

SUPPLEMENTARY MATERIAL

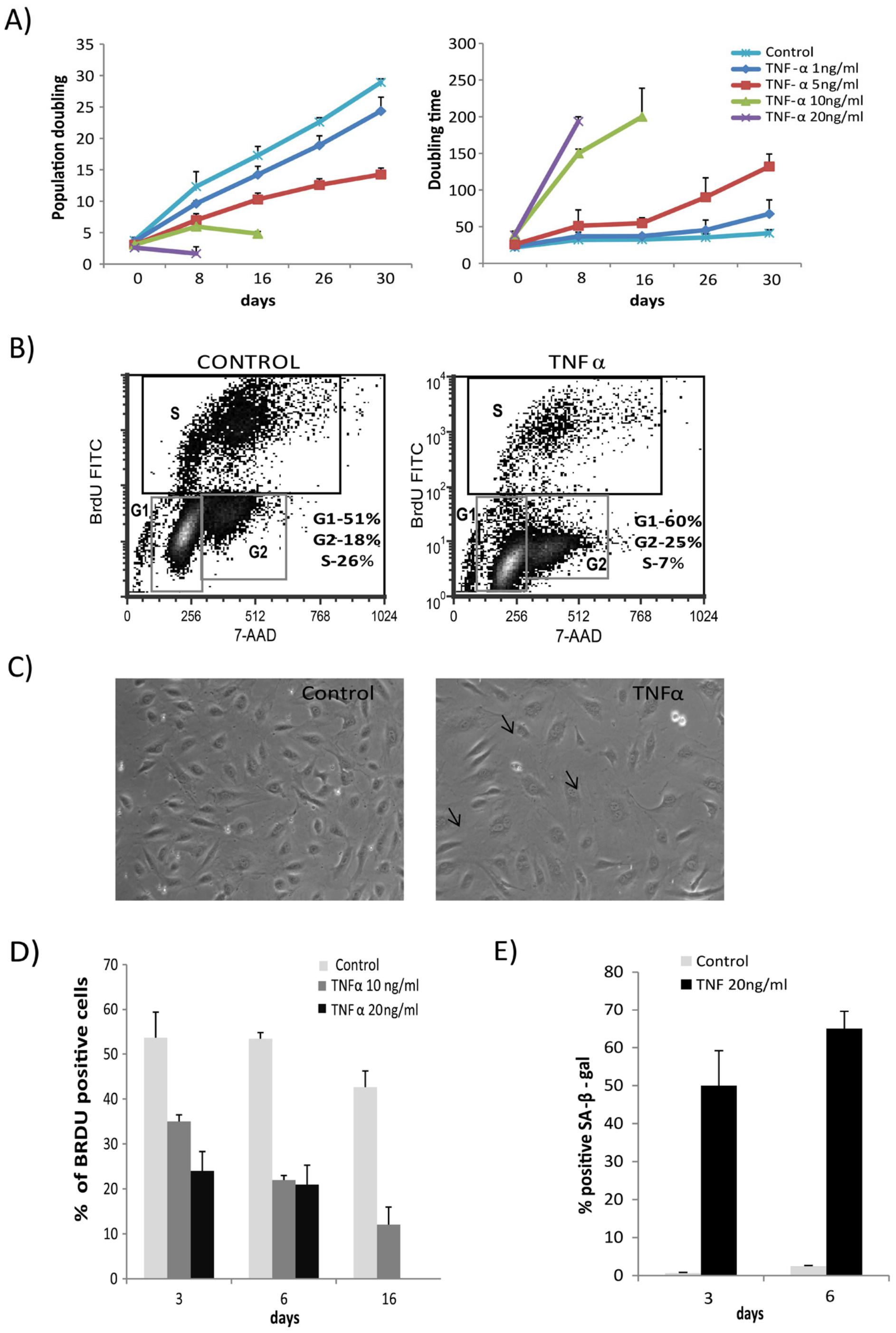

Figure S1. Effects of TNFα on cell proliferation and morphology. (**A**) Growth curve of cells exposed to recombinant human TNFα at different concentrations as indicated. Untreated cells were used as controls. Cumulative population doubling and doubling time were calculated based on cell density at confluence. Data represent mean values from 2 experiments. (**B**) Percentage of gated cells in the S, G1, and G2/M phases in control cells or cells treated with TNFα (5ng/ml) at day 16 detected by FACS analysis. (**C**) Phase contrast photomicrograph image illustrates that cells treated with TNFα (5ng/ml) displayed flattened, enlarged, and bi-nucleated cell morphology compared to control cells. (**D**) The percentage of BrdU-positive cells was determined by FACS in cells untreated or chronically treated with TNFα at the concentrations indicated. Error bars show mean value ± s.d. of 2 independent experiments. (**E**) Percentages of SA-β-gal-positive cells in control or TNFα-treated cultures. The data represent 2 independent counts of 200 cells from 2 independent experiments.

TNF-α induced senescence exhibited a network of SASP

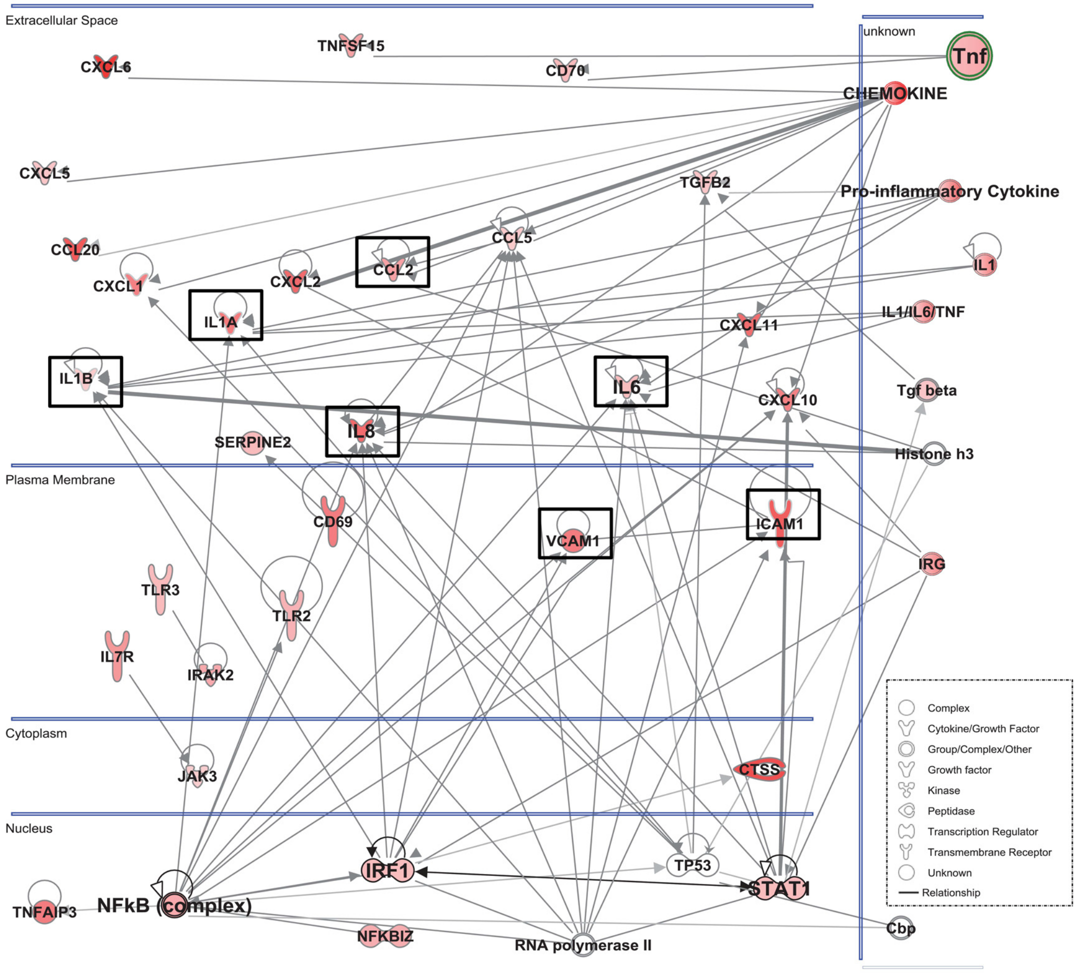

Figure S2. Inflammatory network in TNFα-induced senescence. Network was generated using IPA by importing genes meeting selection criteria (p ≤0.05, ≥ 1.5 fold change) from the TNFα treated group compared to controls. Red symbols represent up-regulated genes, with intensity of node color indicating degree of up-regulation. White indicates genes absent from the list. Continuous lines show direct interactions among molecules and node shape denotes the function of the gene.

TNF-α mediated senescence involves a network of inteferon response genes

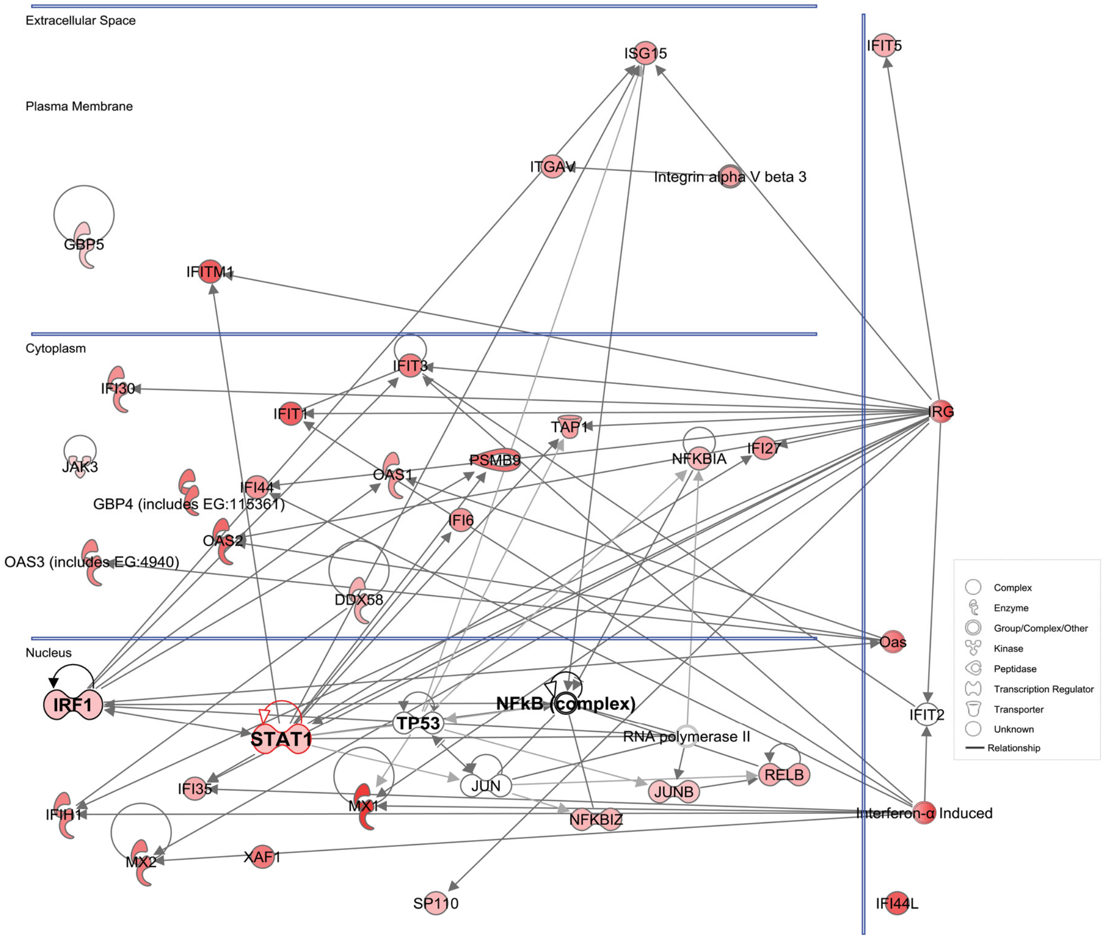

Figure S3. Network of interferon response genes in TNFα-induced senescence. A functional network generated from IPA showing sub-cellular location as indicated. Chronic TNFα treatment differentially up-regulated a cluster of type I and type II interferon response genes relative to controls. Red symbols represent up-regulated genes and the intensity of node color indicates degree of up-regulation. White color indicates genes absent from the list. Shape of nodes denotes the function of gene products.

A)

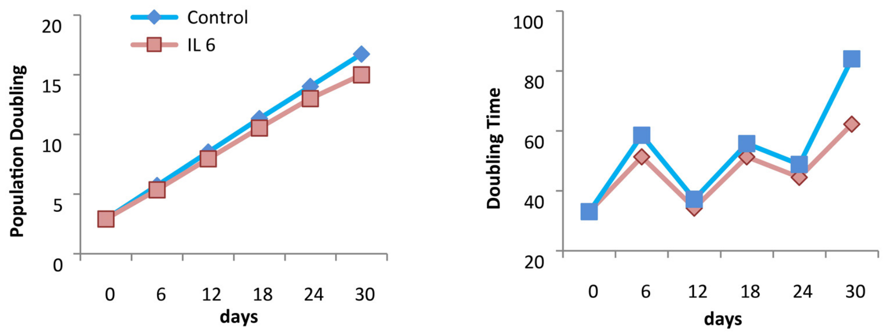

B)

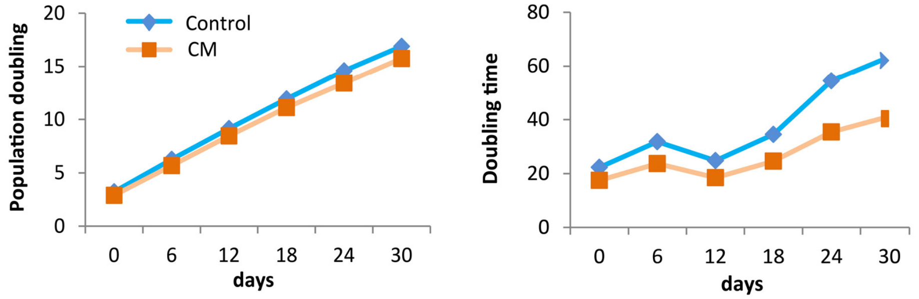

Figure S4. Long-term effects of IL-6 and conditioned medium on HUVECs. (**A**) Growth curve represents population doubling and doubling time of cells chronically treated with human recombinant IL6 (100ng/ml) compared to untreated cells. (**B**) Growth curve of cells treated with conditioned medium from control cells or medium from senescent cells exposed to TNFα (culture media partially enriched [4:1] with conditioned medium).

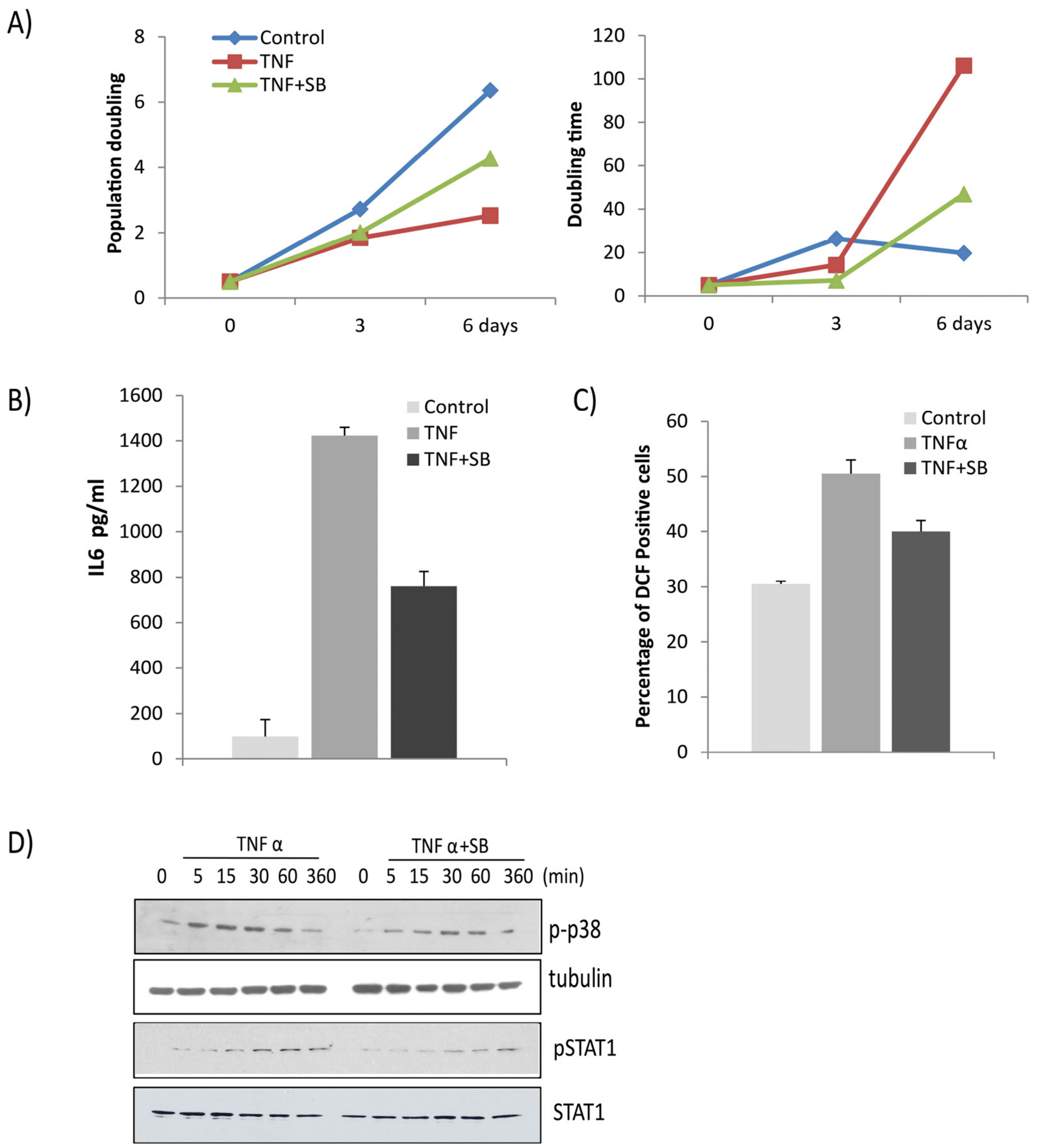

Figure S5. Inhibition of p38 leads to decreased IL-6 secretion, lower ROS levels, and attenuated pSTAT1 activation. (**A**) p38 inhibition partially restores proliferation of TNFα-induced senescent cells. Growth curves of untreated cells or cells treated with TNFα or in combination with 10μM SB203580 (SB) for 48 hours. (**B**) IL6 was quantified by ELISA in medium from cells left untreated or treated with TNFα or in combination with SB. (**C**) FACS detection of ROS levels using DCFDA in cells treated with TNFα alone or TNFα in combination with SB. (**D**) Immunoblot detection of p-p38, p-Ser727-STAT1 and total STAT1 in control cells or cells treated with TNFα alone or TNFα in combination with SB for the indicated times.

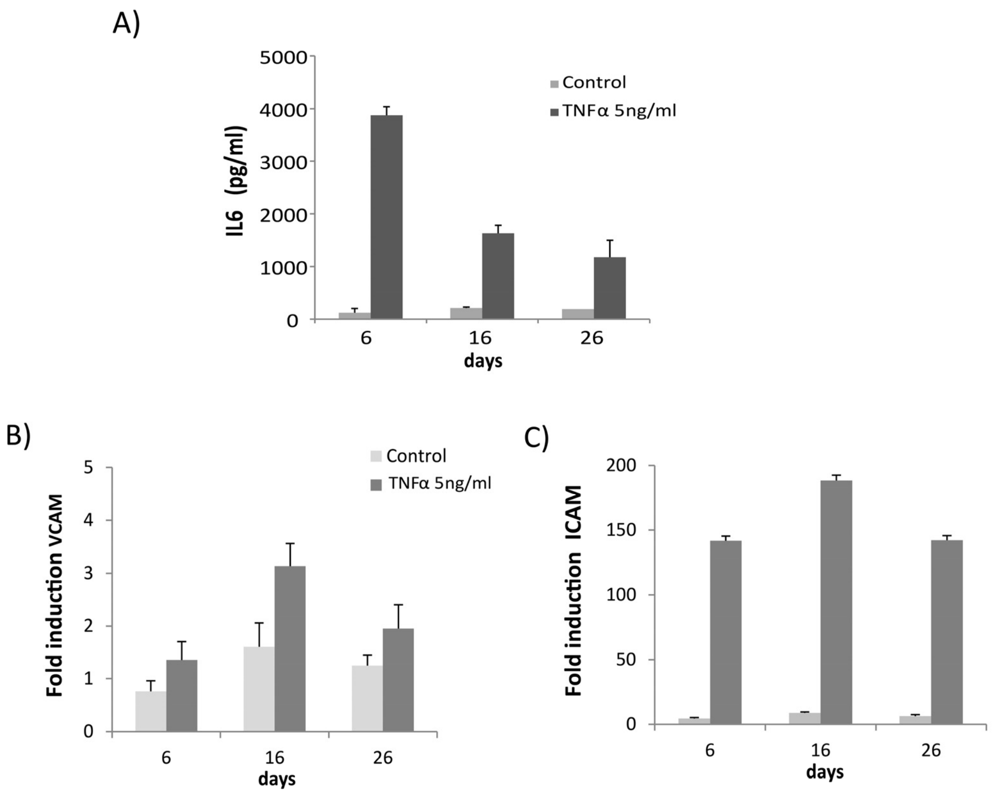

Figure S6. Chronic effects of TNFα on IL-6 secretion and expression of ICAM-1/ VCAM. (**A**) Estimation of IL-6 secretion by ELISA in supernatants from cells left untreated or chronically treated with TNFα (5ng/ml) for the indicated times. Data were normalized to number of cells. (**B**) Cell surface expression of ICAM-1/VCAM was assessed by flow cytometric staining in untreated cells or in cells chronically treated with TNFα (5ng/ml) as indicated. Error bars show mean value ± s.d. of 2 independent experiments.
